# A Persistent Oral Pyogenic Granuloma: A Case Report With Review of Literature

**DOI:** 10.7759/cureus.49326

**Published:** 2023-11-24

**Authors:** Ola Alshuhail, Alaa S Alharbi, Nouf Alakeel

**Affiliations:** 1 Oral Medicine and Diagnostic Sciences Department, College of Dentistry, King Saud University, Riyadh, SAU; 2 College of Dentistry, King Saud University, Riyadh, SAU

**Keywords:** benign tumor, non-neoplastic lesion, soft tissue growth, pregnancy tumor, pyogenic granuloma

## Abstract

Pyogenic granuloma is a non-neoplastic inflammatory reactive hyperplasia commonly found on keratinized tissues caused by different factors such as hormonal imbalance. Pyogenic granuloma has a wide age range and is frequently found in females in the second to third decade. Pyogenic granuloma developed in pregnancy is commonly known as pregnancy tumor. The standard treatment approach is surgical excision of the lesion. In the case report, a 42-year-old female presented with a persistent oral lesion in the left anterior mandible. The lesion first appeared during pregnancy and remained in the oral cavity for two years after delivery. Clinical, radiographic, and histopathological examination revealed a definitive diagnosis of pyogenic granuloma.

## Introduction

Pyogenic granuloma is a non-neoplastic inflammatory reactive hyperplasia commonly found on keratinized tissues caused by chronic irritation, hormonal imbalance, and traumatic injury [1]. It has a wide age range from children to adults, but it is frequently found in females in the second to third decade [1,2].

Pregnancy increases the chance of developing pyogenic granuloma, which is commonly known as pregnancy tumor [1,2]. Clinical presentation of pyogenic granuloma is typically smooth lobulated soft tissue mass pedunculated or sessile, and reddish [2,3]. It is mostly found on the gingiva, and in other sites such as tongue, lip, palate, and oral mucosa [4].

Surgical excision is the most common treatment approach, with a low recurrence rate [1,2]. During pregnancy, some lesions may resolve spontaneously after delivery [2]. Additionally, removing the lesion during pregnancy can increase the likelihood of recurrence [1,2]. Therefore, treatment is typically postponed until after delivery, unless there are esthetic or functional concerns [2,4].

In this case report, a 42-year-old female presented with a persistent oral lesion buccal to the left canine and first premolar. The lesion first appeared during pregnancy and remained in the oral cavity for two years after delivery. A clinical, radiographic, and histopathological comprehensive examination of the lesion was made, which confirmed a definitive diagnosis of pyogenic granuloma.

## Case presentation

**Figure 1:**
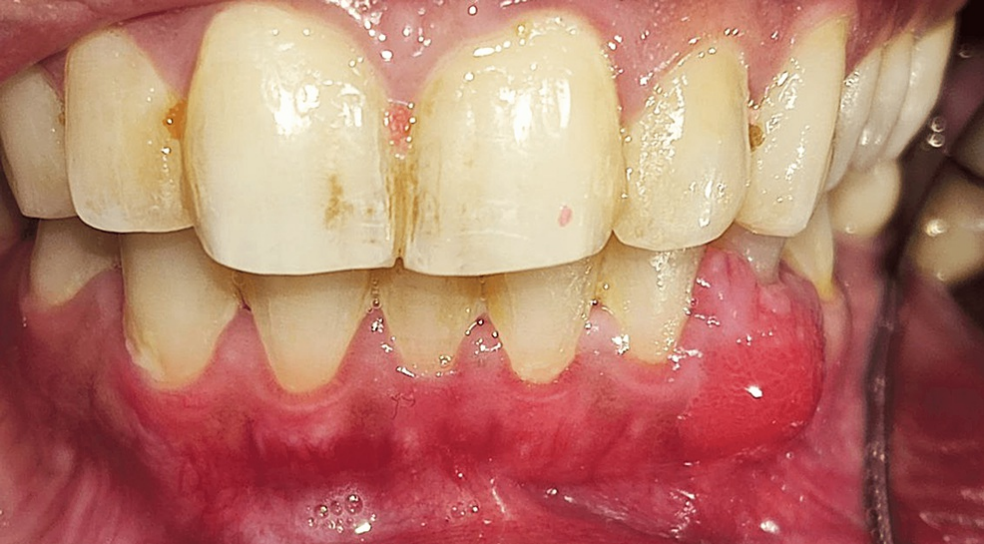
Smooth red sessile lobulated soft tissue mass 2 cm in size labial to the lower left canine and first premolar

**Figure 2:**
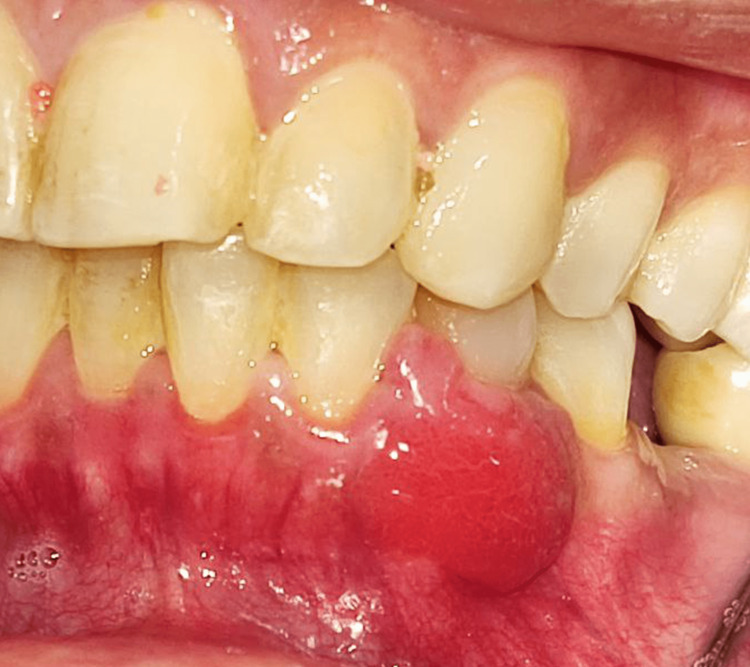
Red sessile soft tissue mass labial to the lower left canine and first premolar

**Figure 3:**
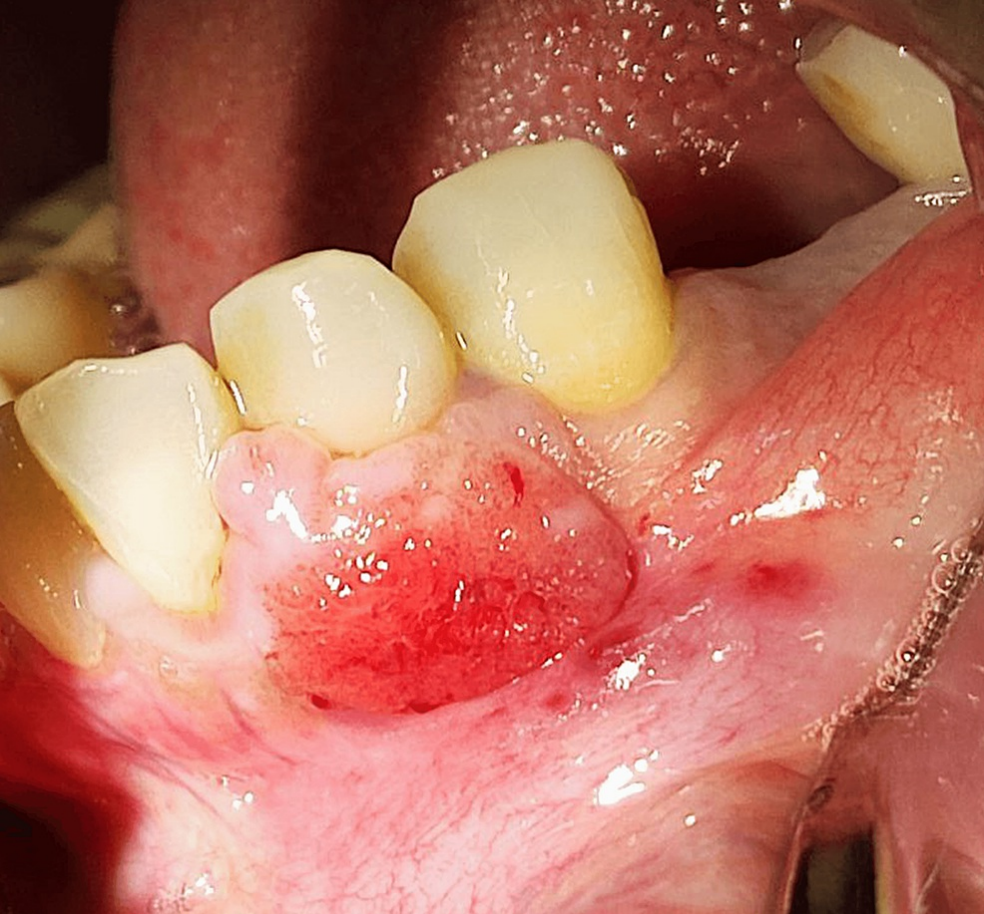
Erythematous ulceration

**Figure 4:**
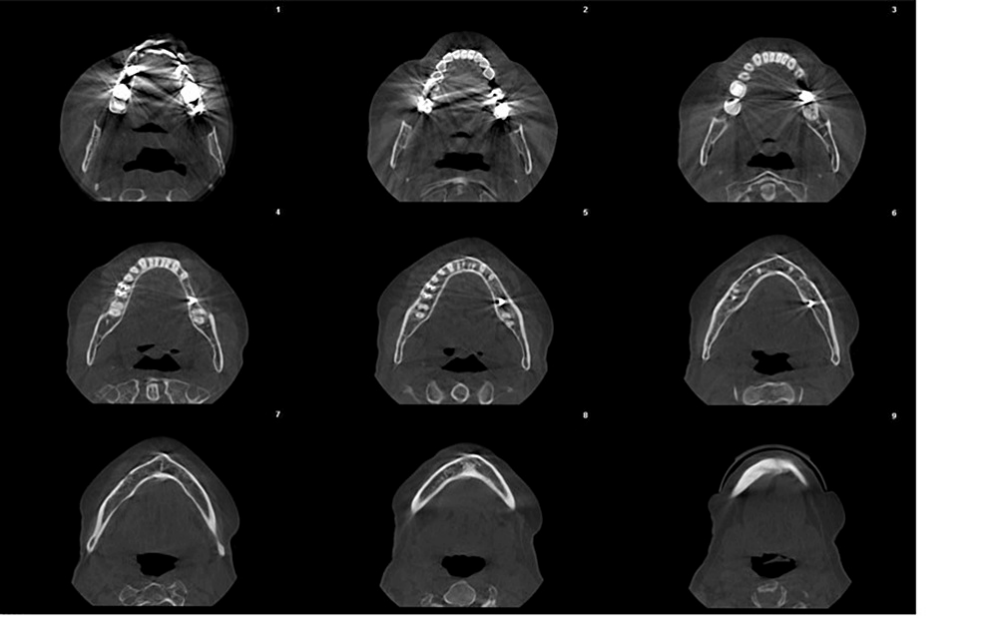
CBCT radiographic investigation showing normal bony structures CBCT, cone beam computed tomography.

**Figure 5:**
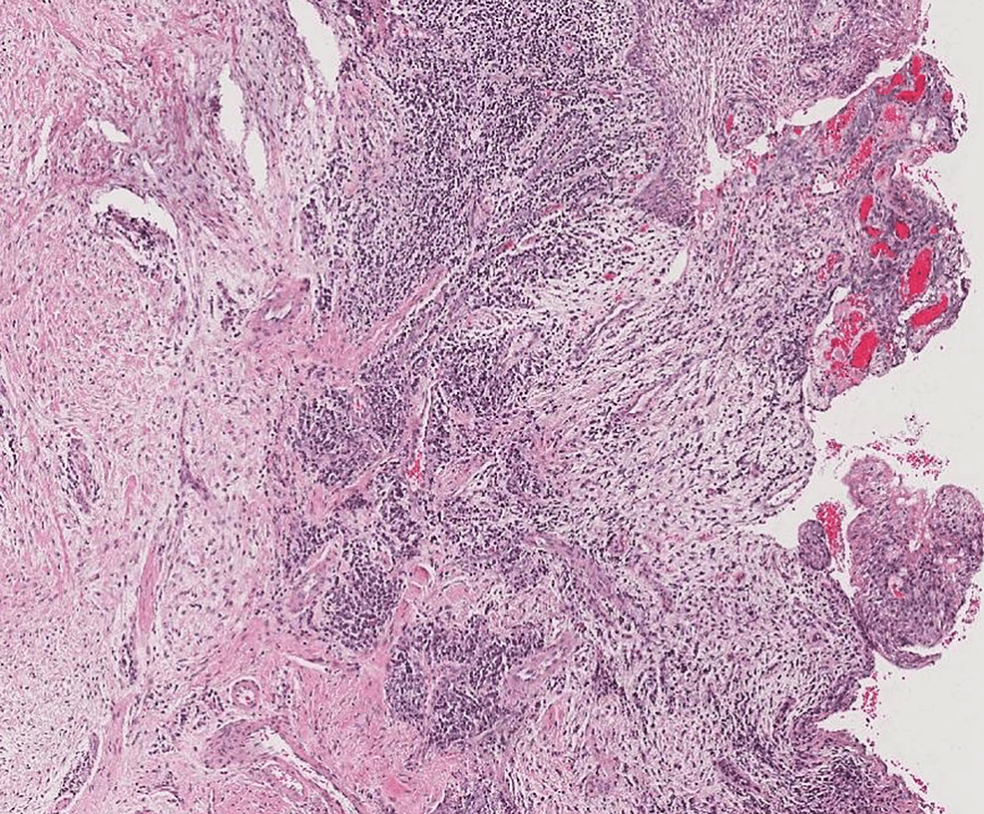
Hematoxylin and eosin (H&E) low-power magnification revealed a non-lobular proliferation of endothelial cells within an inflamed stromal environment with a surface epithelium exhibiting pseudoepitheliomatous hyperplasia

A 42-year-old female patient presented to the Oral Medicine Department of King Saud University - Dental University Hospital in Riyadh, Saudi Arabia, with a painless persistent intra-oral swelling in the left anterior mandible. The lesion first appeared two years ago during pregnancy with a history of enlarged lesion crossing the midline and interfering with mastication. After delivery, the lesion reduced in size and remained persistent in the oral cavity for two years. Clinical examination revealed a 2 cm smooth red lobulated sessile soft tissue mass labial to the lower left canine and first premolar with erythematous ulcerations (Figures 1-3). The associated teeth were vital with no mobility and a probing depth of 5 mm (pseudopockets). Radiographic investigations included cone beam computed tomography (CBCT) examination, which showed normal bony structures and no invasion of the lesion into the bone (Figure 4). A differential diagnosis of peripheral giant cell granuloma and pyogenic granuloma was established. An incisional biopsy of 0.5x0.2x0.1 cm in size was performed to reach the definitive diagnosis. Hematoxylin and eosin-stained (H&E) microscopic examination (Figure 5) revealed a non-lobular proliferation of endothelial cells within an inflamed stromal environment. The surface epithelium exhibited pseudoepitheliomatous hyperplasia. Indications of reactive atypia were present including hyperchromatism, increased nuclear size, and mitotic activity. The microscopic examination is suggestive of pyogenic granuloma.

## Discussion

Pyogenic granuloma is a common tumor-like growth that occurs in the oral cavity [1]. However, the term is misleading since it does not reflect the histological characteristics of a true granuloma, nor does it involve pus or infection. Instead, it is a form of inflammatory hyperplasia that develops in response to local irritation, trauma, or hormonal factors [1,2,4]. Pyogenic granuloma can manifest at any age, but it is more commonly seen in children and young adults [1,2]. It is more prevalent among females, possibly influenced by hormones [1]. During pregnancy, there is an increased likelihood of pyogenic granuloma occurrence, which is referred to as a pregnancy tumor [2,4,5].

Pregnancy tumor develops in 5% of pregnant women due to the high levels of progesterone in pregnancy that increases the response to irritation along with plaque accumulation and gingival inflammation, and it appears in the second to third trimester with common complications of easily bleeding lesion and interference with mastication [5]. In the present case, the patient is in the fifth decade, which is a less common age for a pyogenic granuloma that developed during pregnancy.

Pyogenic granuloma is characterized clinically as a reddish vascular mass that can be smooth or lobulated, and either sessile or pedunculated. It tends to bleed, and it becomes ulcerated in sites subjected to trauma [1-3,6]. The lesion can slowly increase in size over weeks to months, varying from a few millimeters to several centimeters [1,6]. However, the lesion usually does not exceed 2.5 cm in size [1,6].

The most common site of occurrence is the gingiva, accounting for 75% of the cases, followed by the lips, tongue, palate, and buccal mucosa [1,4,7]. It is more frequently found in the maxilla than the mandible, and in the anterior region compared to the posterior [7]. The case presented has typical clinical features except for the location which is in the anterior mandible whereas pyogenic granuloma is commonly found on the labial mucosa of the anterior maxilla [7].

Given the similar clinical features observed with peripheral giant cell granuloma and peripheral ossifying fibroma, performing histopathological examinations is recommended to confirm the diagnosis of pyogenic granuloma [1,8,9]. The histopathological examination of pyogenic granuloma reveals distinctive characteristics of the lesion; it includes a highly vascular proliferation resembling granulation tissue, with the formation of multiple channels of varying sizes [2,7,8]. These channels are engorged with red blood cells and are lined with endothelium, sometimes exhibiting a lobular organization [2,7]. Additionally, there is evidence of an inflammatory cell infiltrate consisting of neutrophils, plasma cells, and lymphocytes [7,8]. Pyogenic granuloma is further subdivided into lobular capillary hemangioma (LCH) and non-lobular capillary hemangioma (non-LCH) varying in the histopathological features in which the LCH type consists of proliferating blood vessels arranged as lobular aggregates whereas the non-LCH type consists of highly vascular proliferation mimicking granulation tissue with mitotic activity that could be seen in the stromal cells in the two types [1].

Radiographic examinations typically do not reveal any specific features related to pyogenic granuloma [2]. However, in rare instances, long-standing gingival pyogenic granuloma can lead to localized alveolar bone loss [2,10]. Moreover, some cases exhibit significant bone loss that resembles malignancy [11]. In our case, a biopsy, along with an intraoral radiograph and CBCT, was conducted to confirm the diagnosis and identify any bone destruction.

The usual treatment for pyogenic granuloma is conservative surgical excision [1,2,7]. During the excision, it is important to extend the removal to the depth of the periosteum and include adjacent teeth in the periphery [1,2,7]. Additionally, any irritants such as calculus or foreign material should be removed [1,2,7]. Other treatment modalities that have been used include cryotherapy, cauterization, and laser therapy [1,12].

The prognosis for pyogenic granuloma is generally favorable [2]. The recurrence rate is approximately 16% and often occurs due to factors like incomplete removal, untreated underlying causes, or repeated irritation and trauma [1,2]. Lesions removed during pregnancy have a higher recurrence rate due to hormonal effects [1,2]. In the absence of esthetic or functional issues, surgical treatment is not recommended as some lesions tend to resolve after delivery [1,2]. Pregnant women should prioritize maintaining oral hygiene and attending regular follow-up appointments [1,4]. However, any surgical interventions should be carefully planned in consultation with the patient's physician [4]. In our case, although the lesion has reduced in size after delivery, it persisted after two years. 

## Conclusions

Pyogenic granuloma is a benign inflammatory reactive hyperplasia that is commonly found on the anterior gingiva of the maxilla with a wide age range of occurrence but frequently with females in their second and third decades. Pregnant women have an increased risk of developing pyogenic granuloma, which is also known as pregnancy tumor. The gold-standard diagnostic investigation is histopathological examination. Surgical excision is the most common treatment approach with a low recurrence rate. Surgical excision of the lesion during pregnancy can increase the risk of recurrence. However, the lesion could be removed in cases of functional or esthetic concerns.
